# Innate Immune System Orchestrates Metabolic Homeostasis and Dysfunction in Visceral Adipose Tissue During Obesity

**DOI:** 10.3389/fimmu.2021.702835

**Published:** 2021-08-06

**Authors:** Yu Zhen, Wentao Shu, Xintong Hou, Yinan Wang

**Affiliations:** ^1^Department of Dermatology, The First Hospital of Jilin University, Changchun, China; ^2^Department of Biobank, Division of Clinical Research, The First Hospital of Jilin University, Changchun, China; ^3^Key Laboratory of Organ Regeneration and Transplantation of the Ministry of Education, The First Hospital of Jilin University, Changchun, China; ^4^National-Local Joint Engineering Laboratory of Animal Models for Human Diseases, The First Hospital of Jilin University, Changchun, China; ^5^Institute of Immunology, Jilin University, Changchun, China

**Keywords:** innate immune system, adipose tissue, metabolism, obesity, inflammation

## Abstract

Arising incidence of metabolic disorders and related diseases caused by obesity is a global health concern. Elucidating the role of the immune system in this process will help to understand the related mechanisms and develop treatment strategies. Here, we have focused on innate immune cells in visceral adipose tissue (VAT) and summarized the roles of these cells in maintaining the homeostasis of VAT. Furthermore, this review reveals the importance of quantitative and functional changes of innate immune cells when the metabolic microenvironment changes due to obesity or excess lipids, and confirms that these changes eventually lead to the occurrence of chronic inflammation and metabolic diseases of VAT. Two perspectives are reviewed, which include sequential changes in various innate immune cells in the steady state of VAT and its imbalance during obesity. Cross-sectional interactions between various innate immune cells at the same time point are also reviewed. Through delineation of a comprehensive perspective of VAT homeostasis in obesity-induced chronic inflammation, and ultimately metabolic dysfunction and disease, we expect to clarify the complex interactive networks among distinct cell populations and propose that these interactions should be taken into account in the development of biotherapeutic strategies.

## Introduction

Overweight and obesity are defined as abnormal or excessive fat accumulation that may impair health. Worldwide, obesity has nearly tripled since 1975. In 2016, 39% of adults aged 18 years and older were overweight, and 13% of adults were obese. In 2019, 38 million children under the age of five were overweight or obese. Obesity, especially abdominal obesity, leads to an increase in associated health complications, such as insulin resistance, type 2 diabetes, and cardiovascular and cerebrovascular diseases (1). Chronic, low-grade inflammation of visceral adipose tissue (VAT) is commonly considered to be a crucial link between obesity and refractory metabolic diseases.

Recently, innate immune cells in VAT have been implicated in the regulation of adipose tissue homeostasis and low-grade inflammation during obesity. Most of such cells are adipose-tissue‒ resident cells that have distinct phenotypes and functions and are altered in numbers or activity during significant changes in the VAT environment (2). Among these cells, VAT-resident innate immune cell types, group 2 innate lymphoid cells (ILC2s), eosinophils, invariant natural killer T cells (iNKTs), and macrophages play key roles in maintaining type II immune state in VAT homeostasis. In obesity, the levels of monocyte chemotactic protein 1 (MCP-1) and leptin are significantly elevated, causing a series of events that mediate chronic inflammation of the adipose tissue. Recruitment, proliferation, activation, or polarization of innate immune cells in VAT shows an overlapping but recognizable timeline. Neutrophils, unique non-VAT-resident cell population, are the first cells to respond to obesity and reach the adipose tissue from the periphery, thereby promoting macrophage aggregation and mediating chronic inflammation. Macrophage proliferation *in situ*, migration of monocytes into adipose tissue, and type I macrophage (M1) polarization lead to the accumulation of macrophages in VAT, which have major pro-inflammatory roles in obesity. Besides, it is accompanied by substantially decreased numbers and altered functions of ILC2s, eosinophils, and iNKTs. Other tissue-resident innate immune cells, such as group 1 innate lymphoid cells (ILC1s), natural killer cells (NKs), and dendritic cells (DCs), which play subordinate roles in the maintenance of VAT homeostasis, also exhibit local proliferation and contribute to a shift from type II cytokine- to type I cytokine-associated immune responses.

In this review, we have focused on the current progress in the fields related to obesity over the last five years. By delineating dynamic and wide-ranging interactions between innate immune cells and VAT environments, we hope to provide a comprehensive and in-depth understanding of the pathogenesis and potential targets of obesity-associated metabolic diseases. In addition, we have described the latest progress related to each cell subpopulation in view of its normal state, functions, and mechanisms in the maintenance of VAT homeostasis. Also, the mechanisms by which each subpopulation responds to lipid stress in VAT and contributes to obesity-associated diseases are explained.

## VAT-Resident Innate Immune Cells

### ILC2s

ILCs are newly identified immune cell types that mirror the phenotypes and functions of T cells, but do not express specific antigen receptors. ILCs include three groups: ILC1s, ILC2s, and ILC3s, which reflect the cytokine profiles of classical CD4-positive helper T cell subsets, for example, Th1, Th2, and Th17 cells. Among these ILCs, the ILC2 population is the most important resident cell population that plays a role in metabolic homeostasis and dysfunction in VAT.

#### ILC2s in VAT Homeostasis

ILC2s play key roles in maintaining a steady state of adipose tissue by secreting type II cytokines to mediate the activation and functional maintenance of downstream VAT-resident regulatory cells, such as eosinophils, iNKTs, and type II macrophages (M2s). ILC2s express IL-33 receptors on their cell surfaces and respond to IL-33 to produce a large number of regulatory cytokines, including IL-4, IL-5, IL-9, IL-13, and IL-33, and play crucial roles in the maintenance of VAT homeostasis (3). The stromal cell marker Sca-1 and platelet-derived growth factor receptor α (PDGFRα)-double positive adipose tissue-resident mesenchymal stromal cells (ATSCs) are dominant IL-33-producing cell populations in both mice and humans (4). In addition to producing IL-33 to induce the activation of ILC2s, ATSCs also support ILC2 proliferation through the intercellular adhesion molecule-1 (ICAM-1)/lymphocyte function-associated antigen-1 (LFA-1) pathway. Subsequently, ILC2-derived IL-4 and IL-13 induce the secretion of C-C motif chemokine 11 (CCL11, also known as eotaxin) from ATSCs to recruit eosinophils (5). Regarding the regulatory mechanism of IL-33 secretion by ATSCs, these cells express IL-17 receptors on their cell surfaces, and IL-17A derived from adipose tissue γδ T cells expands IL-33-secreting ATSCs (6). In addition, approximately 10 to 50% of total ATSCs show surface expression of cadherin-11, and their functions can be modified by either a deficiency or blockade of cadherin-11. Cadherin-11-deficient mice showed higher IL-33 expression in adipose tissue, which resulted in enhanced ILC2 and M2 activation. Although the precise mechanisms for these findings have not yet been clarified, cadherin-11 is speculated to be a potential target for the management of adipose tissue inflammation, diabetes, and metabolic syndrome (7).

ILC2s also mediate type II immune responses by inducing the expansion of regulatory T cells (Tregs). ILC2s express inducible T-cell co-stimulator-ligand (ICOS-L), while Tregs express ICOS on their cell surfaces. Thus, ILC2s induce aggregation of Tregs *via* the ICOS/ICOS-L signaling pathway in VAT (8). IL-33 can also induce increased expression of the co-stimulatory molecule OX40L on the surfaces of ILC2s, which provides co-stimulatory signals to OX40-expressing Tregs and induces expansion of Tregs in adipose tissues (9). Similar to these co-stimulatory signaling pathways, glucocorticoid-induced tumor necrosis factor receptor (GITR), a member of the TNF superfamily, is also an interesting co-stimulatory immune molecule that is expressed on the surfaces of both murine and human ILC2s. GITR engagement on ILC2s *via* a specific agonist can induce Th2 cytokine secretion by activated ILC2s. Interestingly, GITR engagement of ILC2s can also improve glucose homeostasis and protect against established insulin resistance (10).

#### ILC2s in VAT Obesity

Though IL-33 maintains ILC2s function in steady state, it may display complex functions in ILC2s during obesity, which leads to the decreased fraction of ILC2s in VAT The deficiency of ILC2s induces an insufficient production of IL-5, leading to decreased numbers of eosinophils in VAT and decreased secretion of IL-4. In addition to the decreased number of ILC2s, drastic changes in ILC2 functions have attracted widespread attention. In mice that are fed a high-fat diet (HFD), elevated levels of TNF-α mediate the upregulation of PD-1 expression on the surfaces of ILC2s. Meanwhile, type I macrophages (M1s) expressing PD-L1 also increase in numbers and inhibit the secretion of IL-5 and IL-13 by ILC2s through the PD-1/PD-L1 pathway. Depletion of inflammatory macrophages or PD-1 blockade can partially rescue ILC2 functions, which implies that PD-1 could be a target for treatment of obesity-induced metabolic disorders (11).

### Eosinophils

It has been agreed upon that IL-5 and IL-13 secreted by ILC2s induce adipocytes to express CCL11 and CCL24, which are potent attractors of eosinophils, and promote chemotaxis of eosinophils in adipose tissues (12). IL-5 is also critical for proliferation and differentiation of eosinophils *in vivo*. In adipose tissue, eosinophils are often distributed close to M2s and secrete type II cytokines, such as IL-4, IL-10, IL-13, and TGF-β, which participate in anti-inflammatory immune responses and promote M2 polarization and Th2 differentiation. More than 90% of IL-4 in VAT is secreted by eosinophils, and it also functions to maintain insulin sensitivity. Regarding the regulatory roles of eosinophils in macrophage polarization, a recent study has proposed more complex mechanisms. Both eosinophilic EoL-1 cells and mature eosinophils, derived from human cord blood CD34 positive cells, expressed a panel of toll-like receptors (TLRs), among which TLR4 was the most highly expressed TLR. TLR4 ligand-stimulated eosinophils mediate a significant upregulation of M1 markers in cultured monocytes (13). However, since this study has carried out only *in vitro* experiments, *in vivo* experiments are still needed to verify whether these effects and mechanisms occur under physiological conditions.

Recently, the transcriptional repressor Krüppel-like factor 3 (KLF3) has been proposed as an important regulator of the activation and function of eosinophils in adipose tissues. Elevated levels of eosinophil chemoattractants eotaxin-1 and 2 contribute to the increased eosinophil abundance in KLF3-deficient mice, and KLF3 can directly bind and regulate gene expression in adipose tissue-resident eosinophils (14). It is worth noting that only a few studies have examined the roles of eosinophils during VAT homeostasis and obesity in humans. Given the crucial anti-inflammatory functions of eosinophils in mice VAT, studies on the possible protective roles of these cells in obesity in humans and associated metabolic diseases are urgently needed.

### iNKTs

iNKTs express an invariant T cell receptor (TCR) α chain and only recognize lipids presented by CD1d molecules. iNKTs in VAT regulate local homeostasis and obesity through several mechanisms. First, iNKT cells express Fas-ligand (Fas-L), whereas adipocytes express Fas, so iNKT cells exert cytotoxic effects to eliminate unhealthy adipocytes and stimulate differentiation of healthy adipocytes. Therefore, iNKT cells sustain VAT homeostasis in the steady state and also contribute to adipose tissue remodeling in obesity, which may explain why iNKTs are abundantly found close to dead adipocytes (15). In addition, iNKTs play regulatory roles in VAT homeostasis by producing a variety of type II cytokines. iNKTs produce IL-2, IL-4, IL-10, and IL-13 to maintain the normal functions of Tregs and M2s.

A recent study further classified VAT-iNKTs into two subsets according to the expression of NK1.1 on the surfaces of iNKTs cells and revealed that these two subsets control adipose tissue homeostasis through distinct pathways. NK1.1-positive iNKTs produce IFN-γ to drive NK cell-mediated macrophage killing and limit pathogenic macrophage expansion in a lean state. NK1.1-negative iNKTs predominantly produce the anti-inflammatory cytokine IL-10 through the inositol-requiring enzyme 1α (IRE1α)-X-box binding protein 1 (XBP1) pathway as well as the so-called iNKT10 cells. Adoptive transfer of IL-10-producing NK1.1-negative AT-iNKT10 cells can restore metabolic functions in obese mice (16). In addition, a subcutaneous injection of α-galactosylceramide (α-GalCer) administered to HFD-mice can activate VAT-iNKT10 cells and subsequently promote M2 polarization, which may provide a new strategy to improve obesity-induced metabolic diseases (17). Interestingly, another study also reported that IRE1α acts as a metabolic endoplasmic reticulum (ER) stress sensor in obesity and can suppress alternative activation of macrophages. Therefore, IRE1α ablation augments M2 polarization (18). Thus, the IRE1α-XBP1 pathway may play distinct roles in iNKTs and macrophages, and these roles should be taken into consideration when designing this pathway as a potential treatment strategy for obesity-associated diseases. Regarding the distinct phenotype and function of AT-iNKTs, most studies considered it to be a consequence of microenvironment-induced alterations of typical iNKTs. Only one study indicated that the highly conserved feature of iNKT-TCR is essential for the recognition of CD1d-presented ligands that later controls the functional development of AT-iNKTs in the thymus. This suggests that rather than having an induced phenotype, the AT-iNKT cell type is probably a distinct cell type (19).

When mice were fed HFD for 12 weeks, iNKTs, especially iNKT10 cells in adipose tissues, decreased significantly and were mainly present around dead adipocytes (17). Similarly, in morbidly obese patients, VAT-iNKT cells decreased and visceral adipocytes showed increased CD1d expression. As iNKT cells are essential transactivators of many immune cell subsets, iNKT cell reduction in obesity is accompanied by the reduction of other regulatory cells, such as M2s, Tregs, and eosinophils.

The CD1d molecule is expressed in a variety of antigen-presenting cells (APC), such as DCs, macrophages, B cells, and non-hematopoietic cells such as adipocytes and liver cells (20). Although various cell types express CD1d and then activate iNKT cells through TCR-CD1d interactions, CD1d expression in distinct cell types displays different effects. HFD-fed adipocyte-specific CD1d-knockout mice showed severely reduced numbers of iNKT cells in adipose tissues and decreased responses to α-GalCer-induced iNKT activation, which finally aggravates adipose tissue inflammation and insulin resistance (21, 22). Similarly, different CD1d-positive macrophage subsets also contribute to mediate iNKT cell functions in mice. At a steady state, M2s in adipose tissues act as APCs to present antigens to iNKTs. iNKT cell activation by M2s inhibited metaflammation and insulin resistance by promoting Th2 responses and M2 polarization in VAT. HFD can induce a reduction in the expression of CD1d molecules on the surface of AT-M2s, and also cause a change in APCs from M2s to M1s, whereas iNKT cell activation by M1s exacerbate metaflammation and insulin resistance by promoting Th1 responses and inhibiting M2 polarization. Therefore, M2-specific reduction of CD1d expression and alterations of APCs from M2 to M1 are important initiating events that switch iNKT cell-mediated immune responses from anti-inflammatory to pro-inflammatory activities and disrupt the immune balance in VAT of obese mice (23). AT-resident iNKT cells are also innate lipid sensors that play crucial roles in glycemic control and weight regulation in the steady state of VAT *via* the production of fibroblast growth factor 21 (FGF21) (24, 25).

### Macrophages

#### Macrophages in the Maintenance of VAT Homeostasis

Macrophages are the most numerous immune cell types found in VAT. In lean individuals, macrophages are generally considered to be biased towards an M2-like phenotype. IL-4 is required to regulate M2 polarization and activation through several important pathways. The IL-4/IL-4R/Stat-6/peroxisome proliferator-activated receptor gamma (PPAR-γ) pathway is not only necessary for M2 activation, but also important for promoting macrophage survival and inhibiting metabolic inflammation-related cell death (26). In addition, a new IL-4/insulin receptor substrate-2 (IRS2) pathway has been shown to be essential for M2 activation (27). Activation of both pathways is necessary for full activation of M2s.

Several new theories about the roles and mechanisms of macrophages in the maintenance of VAT homeostasis have been proposed recently. First, at a steady state, succinate, as an energetic metabolite of the mitochondrial tricarboxylic acid cycle, induces an anti-inflammatory phenotype in adipose-tissue-resident macrophages through the succinate-succinate receptor 1 (SUCNR1) signaling axis and dampens tissue inflammation. Myeloid SUCNR1-deficient mice showed increased inflammation and glucose intolerance. Moreover, succinate decreased the expression of inflammatory markers in the adipose tissue of lean human subjects but not in that of obese subjects, who had lower expression of SUCNR1 in adipose-tissue-resident macrophages. Therefore, succinate-SUCNR1 signaling can be considered as a metabolic guardian in both the steady state as well as obesity-associated chronic inflammation in VAT (28). However, another study reported discrepant findings. When fed HFD, SUCNR1 knockout mice had reduced numbers of macrophages and crown-like structures in VAT and exhibited significantly improved glucose tolerance. SUCNR1-mediated macrophage chemotaxis aggravated obesity-associated inflammation and metabolic disorders, and this study indicates that SUCNR1 may be an essential therapeutic target in obesity-induced diseases (29). A possible explanation for this discrepancy may be due to the usage of global SUCNR1 knockout mice in the latter study, which could not evaluate the precise role of SUCNR1 in specific cell subsets due to the extensive expression of SUCNR1 in various cell types.

Neuropeptide FF (NPFF), an appetite-reducing neuropeptide, plays an important role in supporting healthy adipose tissue by maintaining metabolically beneficial macrophages. NPFF receptor 2 (NPFFR2) is expressed on the surface of mouse and human VAT macrophages and is upregulated by IL-4. It promotes M2 activation and proliferation *via* enhanced stability of phosphorylated Stat-6 and increased transcription of M2-associated genes (30).

In addition to energy metabolites and neuropeptides, macrophage-derived exosomal microRNAs (miRNAs) have also been demonstrated to modulate insulin sensitivity and glucose homeostasis. VAT macrophage exosomes derived from lean mice can improve glucose tolerance and insulin sensitivity in obese mice. VAT macrophages in obese mice can secrete miRNA-containing exosomes and cause glucose intolerance and insulin resistance when transferred to lean mice. Therefore, different types of miRNA-containing exosomes derived from VAT macrophages may have important regulatory mechanisms in metabolic homeostasis (31, 32). Similarly, M2-polarized bone marrow-derived macrophages (BMDMs) secrete miRNA-690-containing exosomes, which improve glucose tolerance and insulin sensitivity in obese mice (33).

Most recently, exciting insights into the mechanism of metabolic homeostasis in VAT have been uncovered. Adipose-tissue-resident macrophages acquire mitochondria from neighboring adipocytes in a heparan-sulfate-dependent manner. Macrophages that acquire mitochondria from adipocytes appear to regulate and maintain adipose tissue homeostasis. Obesity is associated with decreased transfer of mitochondria from adipocytes to macrophages, which may be due to M1 polarization (34). Taken together, these data suggest that energetic metabolites, neuropeptides, miRNA-containing exosomes, and transferable mitochondria could be new therapeutic targets for obesity-related metabolic disorders.

#### Macrophages in VAT During Obesity

Obesity-induced macrophage alternations include the decrease of M2s and increase of M1s. In lean individuals, VAT macrophages have an alternatively activated (M2) phenotype that limits inflammation and sustains homeostasis. In obesity, a decrease in cell populations, such as ILC2s and eosinophils in VAT leads to insufficient production of IL-4. IL-4 deficiency significantly impairs the differentiation of M2s. Furthermore, IL-4 deficiency also mediates changes in macrophages in obesity through two other essential pathways. First, increased free acids (FA) cause lipotoxicity in the cells of VAT, resulting in cell death and inflammasome activation. The IL-4/Stat-6/PPAR-γ pathway in M2s can protect against lipotoxicity-induced M2 death and inflammasome activation by regulating genes involved in cell survival. Therefore, IL-4 deficiency in obesity increases the susceptibility of M2s to FA-induced cell death (26). In addition, the IL-4/IRS2 pathway is essential for M2 activation in the steady state of VAT, whereas in obesity, hyperinsulinemia inhibits the IL-4/IRS2 pathway by reducing IRS2 expression on macrophages, which finally leads to the impairment of M2 activation (27). However, another study showed that IRS2-deficient macrophages display an anti-inflammatory transcriptional profile (35). Discrepancy between these two studies might be due to the use of different mouse models, which were myeloid cell-specific IRS2-deficient mice and global IRS2-deficient mice, respectively. An important member of the bromodomain and extra-terminal family, Brd4, can cooperate with PPAR-γ in macrophages and regulate the expression of inflammatory cytokines, which contributes to fat accumulation, inflammation, and insulin resistance in VAT during obesity (36).

In obesity, mechanisms related to an increase in M1s include the recruitment of peripheral monocytes toward VAT and differentiation to M1s, as well as proliferation of M1s *in situ*. First, increased serum levels of monocyte chemotactic protein 1 (MCP-1) produced by adipose precursor cells induce the chemotaxis of monocytes from peripheral blood, which depends on the CCR2 or CCR7-mediated pathway (37–39). As early as one week following HFD, an acute reduction in circulating monocyte count, elevated CD11c expression in Ly6c-low monocytes, and macrophage infiltration in VAT occur in mice. Acute reduction of peripheral blood monocyte count might be involved in the early infiltration of macrophages in VAT (40). A classical inflammatory cytokine, IL-6 also plays a critical role in obesity-associated inflammation and diseases. Interestingly, the cell source of IL-6 dramatically affects the physiological metabolic response to IL-6 in mice. IL-6 secreted by adipocytes promotes, whereas IL-6 derived from myeloid cells suppresses macrophage infiltration and M1 polarization in VAT, subsequently inhibiting or improving glucose and insulin tolerance. These opposing functions are due to a switch of IL-6 signaling from a canonical mode (myeloid cells) to a non-canonical mode (adipocytes).Therefore, careful attention should be paid to this phenomenon when developing a blockade to IL-6 as a treatment strategy for obesity-induced disorders (41).

In excessive lipid-loaded condition or obesity, elevated circulating level of TNF-α leads to increased production of leptin. Since multiple immune cell types express leptin receptors, leptin plays a pivotal role in mediating metaflammation during obesity by triggering the aggregation of pro-inflammatory immune cells. However, a study using a leptin-deficient obesity mouse model and leptin administration revealed an unexpected anti-inflammatory role of leptin. Circulating protein and mRNA expression levels of IL-6 and MCP-1, and crown-like structure (CLS) density decreased in VAT by leptin treatment and showed sub-physiological to physiological ranges. Therefore, the effects of leptin on obesity-associated inflammation might be dose-dependent, and the management of obesity-associated diseases by using leptin as a potential therapeutic target requires careful consideration (42).

Both monocyte migration and *in situ* macrophage proliferation contribute to M1 accumulation in VAT during obesity. At an early stage of obesity, while MCP-1, the pivotal monocyte chemoattractant, is not elevated, resident macrophage proliferation *in situ* is significantly enhanced greatly, depending on the IL-4/Stat6 pathway. As obesity progresses, monocyte migration plays a dominant role in macrophage accumulation, and the recruited monocytes further proliferate locally and reside in CLS surrounding dead adipocytes (43). Osteopontin (OPN), a secreted glycoprotein and a migratory cytokine for monocytes and macrophages, was recently reported to not only drive monocyte chemotaxis and macrophage differentiation, but also facilitate local proliferation of macrophages in obesity (44). In addition, adipocyte degeneration in obesity can release cell-free DNA, which promotes macrophage accumulation by promoting MCP-1 expression *via* the TLR-9 pathway. TLR-9-deficient mice show reduced macrophage accumulation and inflammation. Similarly, in humans, plasma DNA levels are significantly higher in patients with visceral obesity and are associated with insulin resistance (45).

The recruited monocytes then polarize into M1s, which exert pro-inflammatory roles by producing cytokines such as IL-1β and TNF-α. TNF-α can further induce increased IL-33-dependent PD-1 expression on the surface of ILC2s, thereby recruiting and activating M1s that express PD-L1. M1-derived pro-inflammatory mediators have been shown to cause metabolic dysregulation and insulin resistance (11).

Obesity is associated with chronic metabolic inflammation and endoplasmic reticulum (ER) stress. Several new mechanistic studies on the polarization of macrophages driven by ER stress have been proposed recently. Firstly, ER-resident transmembrane protein kinase and the most conserved ER stress sensor, IRE1α, have attracted great attention. IRE1α downregulates the expression of interferon regulatory factor 4 (IRF4) and Krüppel-like factor 4(KLF4), which are critical regulators of M2 polarization. Also, IRE1α increases the expression of proinflammatory cytokines in macrophages. Abrogation of myeloid IRE1α does not influence macrophage differentiation or recruitment in the absence of excess nutrition; however, it significantly increases the expression of signature M2-marker genes in HFD-fed mice and protects mice from HFD-induced obesity and metabolic disorders. This finding suggests that IRE1α exerts a suppressive effect on M2 polarization (18). Another study confirmed the role of IRE1α in the regulation of M1/M2 balance. Histone lysine demethylase 6a (Kdm6a) deficiency downregulated IRE1α expression in a demethylase activity-dependent manner and augmented M2 polarization by promoting the secretion of IL-10 (46). However, it should be noted that IRE1α plays opposing roles in iNKTs. IRE1α promoted IL-10 production in NK1.1-negative iNKTs driven by intracellular lipid accumulation, and adoptive transfer of adipose tissue NK1.1-negative iNKTs selectively restored metabolic function in obese mice (16).

Secondly, the transcription factor C/EBP homologous protein (CHOP) has been demonstrated to mediate insulin resistance by modulating macrophage polarization during obesity development. HFD-mediated ER stress induces CHOP upregulation in adipocytes. CHOP deficiency inhibits chronic inflammation and prevents HFD-induced insulin resistance in adipose tissue by enhancing M2 polarization, despite similar amounts of macrophages infiltrating into VAT (47). In addition, IL-29, a member of the type 2 interferon family, has been revealed to play an essential role in metabolic disorders. In HFD-induced obese mice, IL-29 enhanced MCP-1 expression, increased the M1/M2 macrophage ratio, and reduced insulin sensitivity. Similarly, in obese patients, elevated serum IL-29 levels produced by adipocytes upregulated MCP-1 expression and decreased insulin sensitivity. Therefore, IL-29 is thought to play an important regulatory role in obesity-induced inflammation and metabolic disorders by mediating a crosstalk between macrophages and adipocytes (48).

As described above, macrophage-derived exosomal miRNAs can modulate insulin sensitivity and glucose homeostasis. Adipocyte-secreted miRNAs are also involved in obesity-associated metabolic disorders by mediating macrophage polarization. Adipose-selective or adipocyte-specific miR-34a-KO mice showed a significant shift in the polarization of adipose-resident macrophages from proinflammatory M1 to anti-inflammatory M2 phenotype. Accordingly, miR-34a-KO mice were resistant to obesity-induced glucose intolerance, insulin resistance, and systemic inflammation (49). Similarly, HFD also changed the miRNA profile of VAT exosomes by switching the exosomes from an anti-inflammatory phenotype to inflammatory phenotype. The exosomes promoted M1 differentiation partially through the pro-inflammatory miRNA, miRNA-155 (31, 32, 50).

During obesity, in addition to mediating inflammatory reactions in VAT by M1 polarization, macrophages can promote chemotaxis of neutrophils and inflammation *via* the macrophage-neutrophil axis. Adoptive transplantation of macrophages from obese mice can increase the number of neutrophils in the liver and peripheral circulation of a host mouse. Microarray analysis of CD11C^+^ and CD11C^-^ macrophages in VAT isolated from HFD-fed mice also showed an increase in the expression of neutrophil chemotactic genes and protein levels in CD11C^+^ macrophages. In humans, CD11c expression in obese individual adipose tissues is correlated with the expression of neutrophil chemotactic genes (51). Another study found that palmitate, a free fatty acid, can promote macrophages to release nucleotides, which are neutrophil chemotactic factors, through the pannexin-1 channel, a process that may facilitate the recruitment of neutrophils into metabolic tissues during obesity (52).

Monocyte migration, *in situ* macrophage proliferation, and M1 polarization in VAT all contribute to the pro-inflammatory roles of macrophages during obesity. In addition to these aspects, distinct macrophage populations that maintain glucose metabolism and energy balance have been identified recently, including CD301b^+^mononuclear phagocytes (53), mouse Ly 6c^+^ or CD9^+^ macrophages (54, 55), vasculature-associated fat macrophages (56), CD206^+^M2-like macrophages (57), neuropilin-1-expressing macrophages (58), and Trem2^+^ lipid-associated macrophage (59, 60). These studies provide a clearer definition of the cellular and functional heterogeneity of macrophages and provide evidence for the development of new immune-metabolic therapies for the treatment of obesity-related diseases.

## Unique Non-VAT-Resident Innate Cell: Neutrophil

Neutrophils are the most abundant leukocytes present in blood and serve as the first line of defense against microbial invasion by engulfing microorganisms and releasing various enzymes; consequently, neutrophils are unique non-adipose tissue-resident cells. HFD induces the accumulation of neutrophils in VAT through central and peripheral stages. Mice fed HFD for just 1-3 days showed an increased differentiation of myeloid progenitors into neutrophils and monocytes in the bone marrow, and elevated elastase levels in hematopoietic stem and progenitor cells. Elastase depletion prevents neutrophil differentiation and subsequent inflammation and metabolic dysfunction, thus suggesting that HFD rapidly activates myelopoiesis in the bone marrow *via* an elastase-dependent pathway (61). Later, in the peripheral stage, neutrophils increase greatly in the adipose tissue, and the activating marker CD66b shows increased expression. Lipolysis of adipocytes and leukotriene B4 (LTB4) production in adipose tissue induces an accumulation of neutrophils prior to that of macrophages. Subsequently, the interaction of neutrophils with adipocytes induces IL-1β secretion *via* the NF-κB pathway. Furthermore, the LTB4-inflammasome axis contributes to the expression of chemotactic molecules involved in HFD-induced macrophage infiltration into VAT. Rapid accumulation of neutrophils in VAT exerts pro-inflammatory effects through the secretion of IL-1β and TNF-α, and also induces M1 macrophage aggregation (62).

Interestingly, two recent studies have reported that VAT macrophages can also recruit neutrophils during obesity. One study using microarray analysis of adipose tissue macrophages showed that obesity resulted in enhanced expression of neutrophil chemotaxis genes, specifically in CD11c^+^macrophage. In humans, CD11c expression in the VAT of obese individuals correlates with the expression of neutrophil chemotactic genes in VAT (51). Another study found that palmitate, a saturated fatty acid, can upregulate pannexin-1 channels in macrophages, and also found that macrophages attract neutrophils into metabolic tissues during obesity by releasing nucleotides (52). Therefore, neutrophils recruited to the adipose tissue during obesity induce macrophage aggregation. In turn, macrophages can also mediate chemotaxis and aggregation of neutrophils, thereby forming a neutrophil-macrophage positive feedback mechanism and participate in obesity-mediated diseases.

In addition to accumulating at an early stage in VAT, promoting macrophage aggregation, and mediating chronic inflammation in obesity, neutrophils in obese diabetic mice also display functional defects, such as decreased phagocytic ability and reactive oxygen species (ROS) production, which explains the increased incidence of recurrent, chronic infection in obese individuals. Administration of exogenous granulocyte-macrophage colony stimulating factor (GM-CSF) can enhance neutrophil phagocytosis and ROS generation ability to improve infection outcomes (63). Another study confirmed the functional defects in neutrophils in HFD-induced obese rats. In this study, neutrophils derived from obese and diabetic rats showed significant tolerance to lipopolysaccharide administration due to the impaired production of cytokines and chemokines. Furthermore, the neutrophils showed increased cell death and impaired migration activity (64).

However, in recent years, there has been little research in this area in humans. Cell counts of classical monocytes and neutrophils are associated with both body composition and metabolic parameters in obese children, suggesting that these cells may play critical roles in obesity-induced disorders (65). Another study also demonstrated a higher frequency of neutrophils in the peripheral blood of obese children group compared to children of normal weight group. Obesity altered the correlation profile of the expression of costimulation, recognition, and activation molecules by neutrophils. In addition, in the obese children group, neutrophils showed an increased expression of pro-inflammatory cytokines, such as IL-6, IL-12, IL-1β, and TNF-α, while showing decreased expression of IL-10, which suggests that neutrophils in childhood obesity exert pro-inflammatory effects and contribute to obesity-associated disorders (66).

## Other Innate Cell Populations

### ILC1s

In humans, ILC1s, in contrast to NK cells, lack cell surface markers such as CD56, CD16 and CD94, as well as the transcription factor eomesodermin (Eomes). Also, ILC1s do not express cytotoxic molecules such as perforin and granzyme B. The classification of ILC1s is complex because of their distinct tissue distribution, marker expression, transcription profiles, and different functions. NK1.1^+^CD3^-^c-kit^-^ retinoic acid receptor-related orphan receptor-γt^-^ (RORγt^-^) cells are identified as AT-ILC1s and constitute 22–30% of adipose lymphocytes. Similarly, CD56^+^CD3^-^c-kit^-^ROR-γt^-^ cells are identified as human AT-ILC1s and are enriched in VAT comprising about 7-20% of lymphocytes. Interestingly, ILC1s can kill macrophages derived from lean mice *ex vivo* in a dose-dependent manner, and ILC1s kill M2s more effectively than M1s. This is the first study to put forward a new mechanism by which macrophages, especially M2s, are kept in check by cytotoxic adipose tissue ILC1s in lean mice. Obesity leads to a loss of the cytotoxic function of AT-ILC1s, leading to accumulation of proinflammatory macrophages and metabolic disorders (67). Another study used a transcription factor, Eomes, and integrin CD49b (also known as DX5) to identify ILC1 subsets in mouse adipose tissues. DX5^-^Eomes^-^ ILC1s were identified as a unique population of phenotypically stable and functionally distinct adipose-resident ILC1s that maintain long-term tissue residency. Excess energy drives a nearly production of IL-12 in VAT and leads to the proliferation and accumulation of AT-resident ILC1s, which depends on the IL-12/Stat4 pathway. Using parabiosis obesity mouse models, it was shown that ILC1 infiltration from the periphery is a primary source of increased AT-ILC1 populations during obesity, and proliferation of ILC1s also contributes to increased numbers of AT-ILC1s. Elevated levels of IFN-γ produced by ILC1s drive proinflammatory macrophage polarization and finally lead to obesity-related disorders (68).

Similar changes and mechanisms also exist in humans. Patients with type 2 diabetes (T2D) display greater numbers and frequencies of AT-ILC1s compared to normal individuals (69). Human AT-ILC1s are also involved in the development of T2D by promoting adipose tissue fibrogenesis and CD11c^+^ macrophage activation (70). Also, an adaptor protein, Lnk, can constrain proliferation and activation of AT-ILC1s by regulating cell reactivity against IL-15, thereby triggering proinflammatory M1 accumulation. Therefore, Lnk plays essential roles in mediating glucose tolerance by inhibiting AT inflammation and insulin resistance, and could be a possible therapeutic target for obesity-induced diseases (71).

### NKs

NKs are important innate cell populations that defend against pathogens and tumors without a need for prior sensitization. In VAT of lean mice, adipocytes show a low expression of NK cell-activating receptor 1 (NCR1) ligands. After four weeks of HFD, adipocytes in VAT show an increased expression of ligands for NCR1, which activates NK cells to produce IFN-γ and drive M1 macrophage differentiation. Selective NK cell depletion or NCR1 deficiency reduces macrophage accumulation, decreases pro-inflammatory gene expression, and delays systemic insulin resistance (72). In addition, IL-15, which is required for NK cell development, homeostasis, and function, increases in VAT during obesity and induces the proliferation and aggregation of NKs in adipose tissue. Administering IL-15 to expand NKs increases the number of VAT-M1s and exaggerates VAT inflammation and insulin resistance (73).

A recent study further identified a distinct NK cell population derived from the VAT of HFD-fed mice, the induction of which depends on the IL-6/Stat-3 pathway. Four-fold upregulation of the IL-6Rα gene and seven-fold upregulation of the colony-stimulating factor 1 receptor gene discriminate this special NK cell type from classical NKs. Depletion of this distinct NK cell population reduced obesity and improved glucose metabolism in HFD-fed mice. Therefore, this distinct NK cell population contributes to obesity-associated inflammation and metabolic disorders in mice and humans, thus providing a potential target for the treatment of obesity-induced diseases (74).

As classical cytotoxic immune cells, NKs are capable of carrying out potent cytotoxic actions against tumor cells, virus-infected cells, and stressed cells. Recent studies have revealed that obesity can also alter the normal physiological functions of NKs. In humans, a higher BMI is accompanied by increased activation of peripheral NKs, manifested by increased expression of CD69 molecules and increased levels of granzyme B. However, the ability of these activated NKs to degranulate and produce cytokines and chemokines decreases, indicating that in obesity, NKs react abnormally and their ability to eliminate target cells is weakened. Therefore, obese individuals are more likely to develop cancers or infectious diseases (75). In obese conditions, inducing the metabolic reprogramming of NKs by administration of IL-15/IL-15Rα leads to the expansion and maturation of AT-NKs and improves the prognosis of tumors (76). Similarly, compared with normal children, obese children show enhanced NK cell activation and metabolism, such as PD-1 expression, mammalian target of rapamycin activation, extracellular acidification rate, and mitochondrial reactive oxygen species, but their ability to respond to external stimuli reduces, eventually leading to the loss of NK cell proliferation and tumor killing function. Therefore, childhood obesity has a negative impact on anti-tumor immunity. Nevertheless, NK cell functions can be reactivated by weight loss in obese adults (77).

A study on the mechanism of obesity-induced defects in NK cell function showed that obesity induces PPAR-driven lipid deposition in NKs, causing NK cell metabolism and traffic paralysis, which leads to impaired anti-tumor activity of NKs. Inhibiting the PPAR pathway or blocking lipids from entering the mitochondria can reverse the metabolic paralysis of NKs and restore their cytotoxic functions (78).

There are many studies on the role of AT-NKs in obesity-induced disorders and the influence of obesity on physiological functions of AT-NKs in both humans and mice; however, the main limitation of human studies was that only NKs in peripheral blood, but not in adipose tissue, were examined. Moreover, in addition to an increase in the number of NKs, their functions were also altered, including enhanced pro-inflammatory functions and weakened killing functions, which directly affected anti-tumor activity. Using IL-15 to amplify AT-NK and to promote an anti-tumor effect also aggravated inflammation and insulin resistance in adipose tissues. Therefore, we need to comprehensively consider various conflicting factors when adjusting the numbers and functions of NKs as an obesity treatment strategy and adjust them accurately.

### DCs

DCs are specialized APC that regulate lymphocyte-mediated immune responses. DCs are divided into two subtypes: CD11c-positive conventional DCs (cDCs) (identified by a high expression of MHC II and hence have a strong ability to capture and present antigens and CD123-positive plasmacytoid DCs (pDCs) (which play essential roles in anti-viral responses). Under physiological conditions, almost all DCs in VAT exist as cDCs and maintain a tolerogenic phenotype. In the case of over nutrition, cDCs in adipose tissue increase in numbers and the signaling pathways that exert anti-inflammatory effects are blocked, which promotes adipose tissue inflammation (79). Circulating pDCs are recruited to adipose tissue during obesity, leading to metabolic disorders (80). The following sections review the roles and mechanisms of different DC subgroups in a steady state of adipose tissues and obesity-associated conditions.

#### cDCs

As AT-macrophages (ATMs) and AT-DCs share the common surface markers CD11c and CD11b, surface expression of CD64 can be used to distinguish between ATMs and AT-DCs, based on the results of gene analysis. CD45^+^CD11c^+^CD64^-^ cells are identified as AT-DCs, whereas CD45^+^CD64^+^ cells are considered as ATMs. In addition, HFD-induced ATM accumulation in monocytes is dependent on CCR2, whereas AT-DC accumulation is less dependent on CCR2 but more dependent on CCR7, suggesting that AT-DCs play an independent role in VAT inflammation during obesity. In addition, CD45^+^CD64^-^CD11c^+^ AT-DC and CD45^+^CD64^+^ ATM have also been identified in obese human adipose tissues (37). Another study had a different opinion regarding the dependency of ATM accumulation on CCR7 in obesity. Obesity promoted the accumulation of CD11c^+^ cells, including AT-DCs and ATMs, in the adipose tissue. CCR7-deficient obese mice showed reduced accumulation of CD11c^+^ cells, including AT-DCs and ATMs. Therefore, this study suggests that therapeutic administration of anti-CCR7 antibody is a feasible approach for treating obesity-associated metabolic diseases (38). A possible explanation for this discrepancy is that ATM accumulation may be indirectly dependent on CCR7. The reduction in ATMs is a consequence of decreased CCR2 levels due to decreased accumulation of CD8^+^ T cells in CCR7-deficient obese mice.

A recent study reported the key mechanisms by which cDCs retain an anti-inflammatory phenotype in the steady state of VAT, and how VAT-cDCs switch to the pro-inflammatory state in obesity. cDCs can be categorized into two subsets *via* different cell surface molecule expression: cDC1s express CD8 and CD103, while cDC2s express CD11b. These two subsets play regulatory roles *via* different transcription factor-induced signaling pathways: the cDC1 subset shows upregulated Wnt/β-catenin pathway and promotes anti-inflammatory responses *via* enhanced production of IL-10; the VAT-cDC2 subset upregulates the PPARγ pathway and inhibits inflammation by mediating the NF-κB signaling pathway (79). Furthermore, the same group demonstrated that constitutively activated β-catenin pathway in cDC1s can provide healthier immune and metabolic phenotypes in islets, which enables the islets to secrete more insulin in response to over nutrition conditions. When obesity occurs, adipocyte hyperplasia decreases the availability of β-catenin ligands for cDC1s; at the same time, the expression of PPAR-γ in cDC2s is also reduced, leading to pro-inflammatory immune responses and obesity-associated disorders (81).

#### pDCs

pDCs play a major role in anti-viral immune responses characterized by a rapid production of type I interferon. Recently, exciting insights into the involvement of pDCs in obesity-associated metabolic perturbations have been uncovered. Chemerin, a chemokine that regulates adipocyte development, differentiation, and metabolic functions shows elevated systemic levels in obese patients with metabolic syndrome. Chemokine-like receptor 1 (CMKLR1), a receptor for chemerin, is expressed specifically on the surface of pDCs in VAT. During obesity, VAT-derived chemerin can recruit pDCs through CMKLR1, and neutralization of CMKLR1 abolishes chemerin-induced pDC migration. After the recruitment of pDCs, high-mobility group B1 protein (HMGB1) and extracellular self-DNA molecules produced by adipose tissues trigger TLR-9 activation in pDCs, leading to type I IFN production *in situ*. Subsequently, type I IFN promotes pro-inflammatory polarization of AT-resident macrophages and aggravates adipose tissue inflammation and consequent metabolic disorders in obesity (80). Similar functions and mechanisms have also been confirmed in humans. pDCs and type I IFN signaling can carry out critical functions in obesity-associated metabolic complications in mice. An experiment using pDC or IFN-α-receptor-deficient mice demonstrated that obesity and insulin resistance are related to the recruitment of pDCs to adipose tissues, and that pDCs regulate metabolism and promote obesity through the type I IFN signaling pathway (82).

## Summary

**Figure 1:**
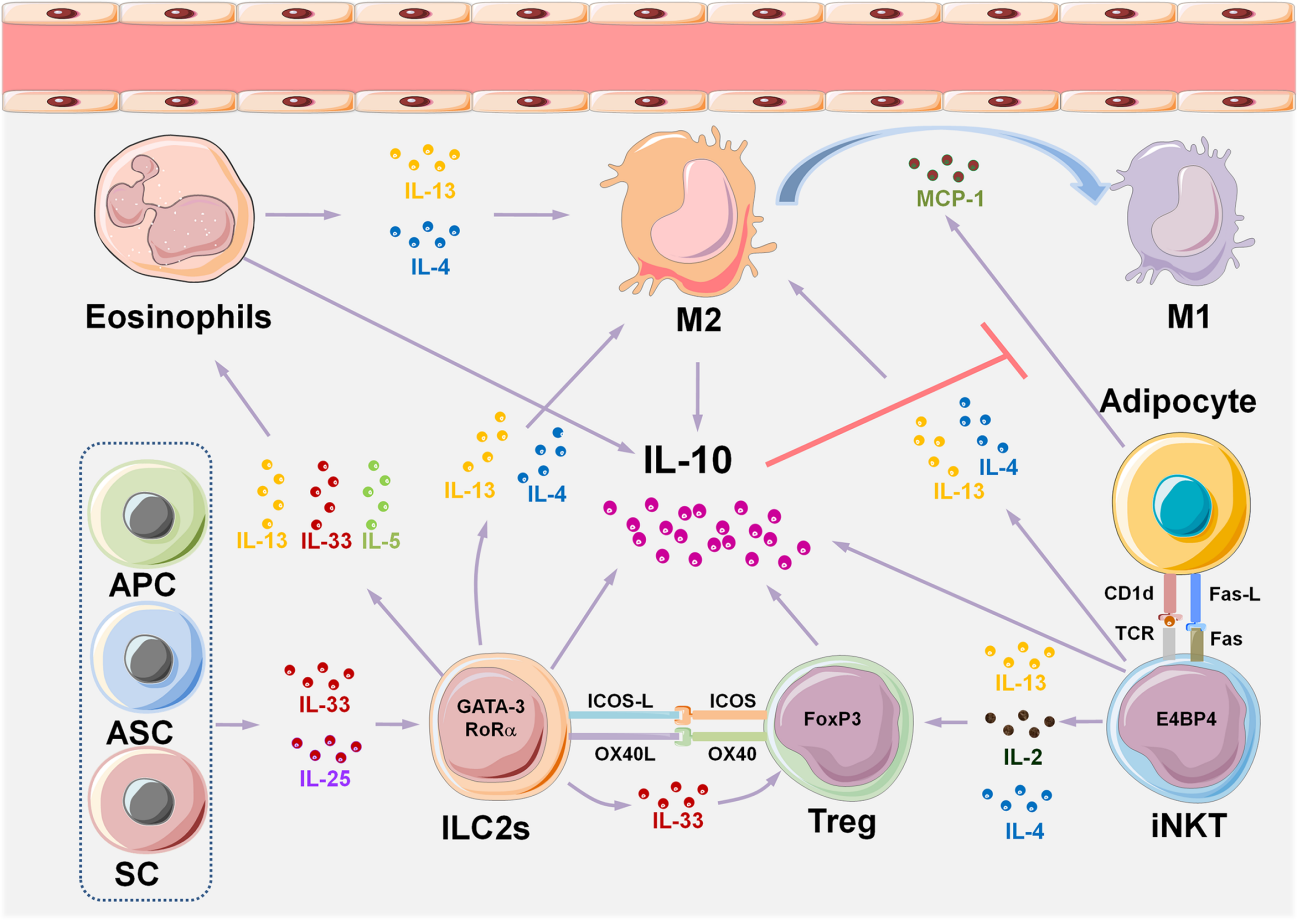
VAT immune cell composition and multiple interactive regulatory networks in metabolic homeostasis. A variety of tissue-resident innate immune cells are distributed in VAT, either interacting with each other or with adipocytes to maintain metabolic homeostasis by producing cytokines. The IL-33/ILC2s pathway plays an initial and key role in maintaining type II immune state in VAT homeostasis. Eosinophils, M2, iNKTs, and Tregs also play a role in maintaining a steady state of VAT. IL-33 is considered to be an initial cytokine that induces and sustains the functions of ILC2s, eosinophils, M2, and Tregs. IL-10 is the core effector cytokine that maintains type II immune response in normal VAT, and almost all the VAT-resident cell types, such as eosinophils, M2, iNKTs, and Tregs produce IL-10. The critical role of IL-10 is to inhibit the production of MCP-1 by adipocytes, thereby reducing the accumulation of monocytes in adipose tissue, which prevents their polarization into pro-inflammatory M1. IL-10-producing iNKT cells uniquely express the transcription factor E4BP4 that controls transcription of the gene encoding IL-10 in Tregs, and differ from conventional iNKT cells which express PLZF. APCs, adipose progenitor cells; ASCs, adipose stem cells; SCs, stromal cells; ILC2s, group 2 innate lymphoid cells; VAT, visceral adipose tissue; M2, type II macrophages; M1, type I macrophages; iNKTs, invariant natural killer T cells; MCP-1, monocyte chemotactic protein 1; Tregs, regulatory T cells; E4BP4, E4 promoter-binding protein 4; PLZF, promyelocytic leukemia variant fusion genes.

**Figure 2:**
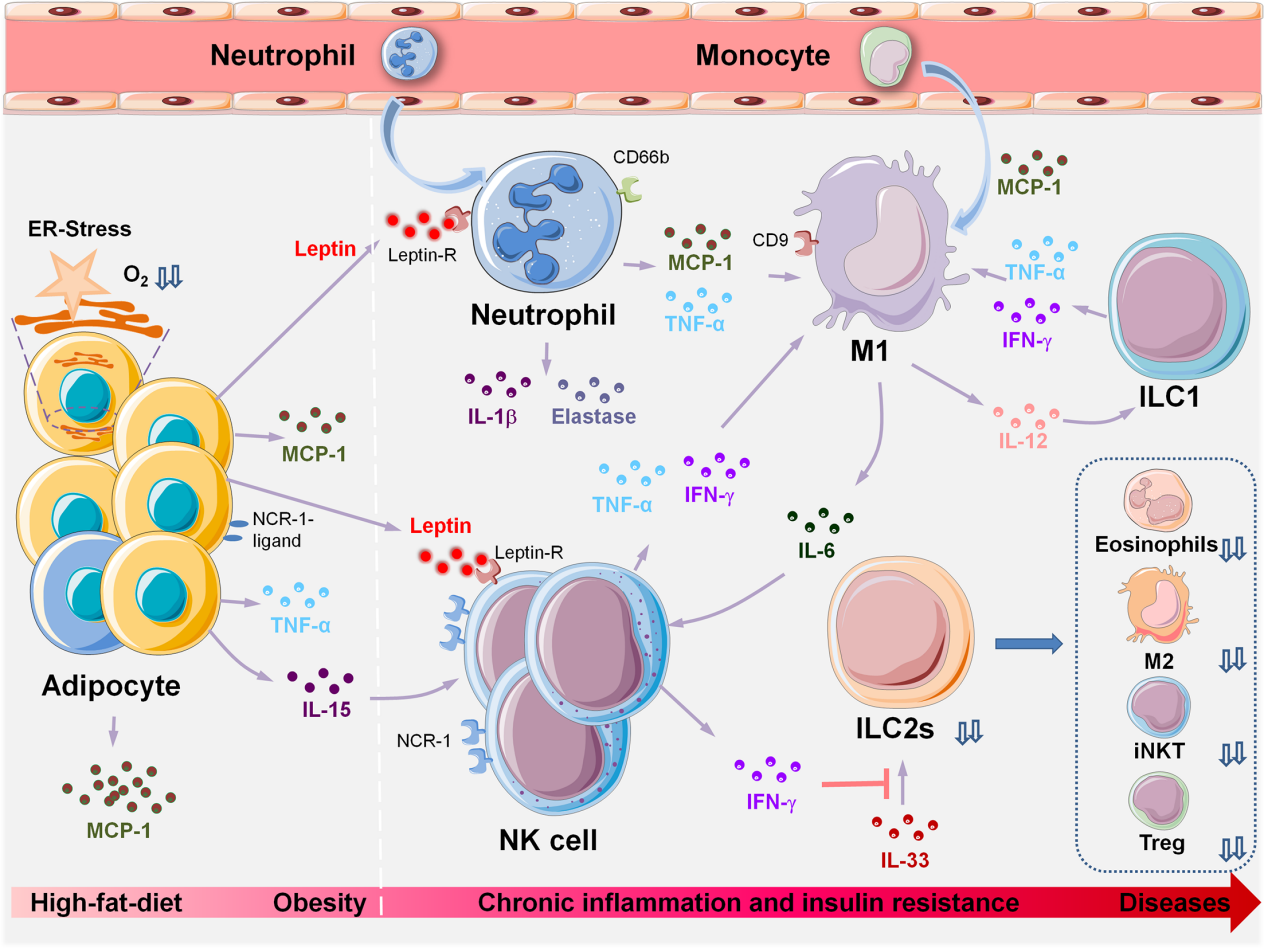
Innate immune cell populations orchestrate metabolic dysfunction in VAT during HFD-induced obesity and associated diseases. HFD-induced obesity leads to adipocyte hypertrophy, pyrolysis, and necrosis. Changes in VAT microenvironment and increased oxygen consumption lead to hypoxia, lipid spillover, and ER stress. Adipocytes and precursor cells produce large amounts of MCP-1 and leptin. MCP-1 induces chemotaxis in a large number of monocytes from peripheral blood, and more than 90% of recruited monocytes locally polarize into M1, which induces and sustains the inflammatory state of VAT. Increased levels of leptin are induced by the inflammatory cytokine TNF-α. Neutrophils, macrophages, NK cells, and activated T and B cells express leptin receptors on their cell surfaces; hence, leptin may be the initial trigger that causes aggregation of these inflammatory cells during obesity, leading to a VAT inflammatory response. The following events occur in the following chronological order: peripheral immune cells (neutrophils, NK) are recruited to the local adipose tissue. Obesity induces upregulation of ligands of NCR-1 on the surface of adipocytes and stimulates NK cell proliferation. Next, monocytes are recruited to the local adipose tissue by MCP-1 and differentiate into M1. IFN-γ can inhibit the reactivity of ILC2s with IL-33, and the deficiency or dysfunction of ILC2s results in insufficient production of IL-4, 5,10,13,33, thus leading to significant decreases in the number of eosinophils, M2, iNKTs and Tregs. VAT, visceral adipose tissue; HFD, high-fat diet; ER-stress, endoplasmic reticulum stress; MCP-1, monocyte chemotactic protein 1; M1, Type I macrophages; NCR-1, NK cell-activating receptor-1; iNKTs, invariant natural killer T cells; Tregs, regulatory T cells; ILCs, innate lymphoid cells.

Through delineation of the panoramic picture of visceral adipose tissue homeostasis (**Figure 1**) related to obesity-induced chronic inflammation (**Figure 2**), and ultimately metabolic dysfunction and disease, we can elucidate initial factors, key factors, or events associated with these pathophysiological processes. Collectively, in the maintenance of VAT metabolism homeostasis, the key initial cytokines and innate immune cells are IL-33 and ILC2s, and the most important effector cytokines are IL-4 and IL-10. IL-33 mediates the activation of ILC2s and Tregs in resting adipose tissue, and ILC2s produce IL-4, IL-5, and IL-13, which further induce aggregation of the most critical immune-modulator cells: M2s and Tregs. Similar to the core roles of IL-33, ILC2s, IL-4, and IL-10 in the maintenance of homeostasis, MCP-1 and leptin play initial and critical roles in obesity-induced chronic inflammation through the recruitment of monocytes, which then polarize into M1s, and also *via* the aggregation of other inflammatory immune cells. A comprehensive and thorough understanding of these key events and factors will help in the development of biotherapeutic strategies toward these targets in obesity-associated diseases.

Because of phenotypic flexibility and functional diversity, macrophage is an ideal therapeutic target for obesity-associated diseases. As described above, PD-1 blockade, subcutaneous injection of α- GalCer, and ablation of IRE1α promoted M2 polarization and improve obesity-induced metabolic diseases. Recently, the most attractive approaches to modifying macrophage polarization are the miRNA-containing exosomes and transferable mitochondria due to advances in potential delivery systems. However, when developing treatment strategies for obesity-related diseases based on key factors or cells during obesity, it is still necessary to have a clearer understanding of the following factors. First, as various cells and molecules show conflicting, redundant, or multiple effects (such as IL-10, leptin, ILC2s, different subtypes of macrophages, and so on, as described above), further elucidation of interactive networks in obesity-associated diseases is needed to develop precise and individualized treatment strategies.

Furthermore, most of the studies in this field use HFD-induced obesity or diabetes mellitus mouse models, such as db/db or ob/ob mice as basic experimental platforms, and also employ various conditional gene knockout or transgenic mice to accomplish loss or gain of function experiments to clarify specific biological functions in mice. There are several crucial differences in the immune systems of humans and mice. A few studies have made a significant progress towards understanding real-world events and mechanisms in humans; however, they still have the following limitations: (1) for ethical reasons, human visceral adipose tissues have a lower feasibility and accessibility to be used for research purposes, and compromised subcutaneous adipose tissues or human adipocyte cell lines may not resemble the distinct characteristics of visceral adipose tissues; (2) human samples can only be obtained at specific time points; consequently, it is not ideal to simulate a real dynamic pathophysiological process. Therefore, using a humanized animal model that mimics the human immune system to observe actual and traceable onset of human obesity-induced disorders may provide more valuable insights towards developing promising strategies for obesity-related diseases in the future.

## Author Contributions

All authors contributed to the article and approved the submitted version. YZ and WS performed bibliographical research and co-wrote the manuscript. XH participated in paper writing and figure design. YW conceived the idea of the review, provided comments, and reviewed the manuscript.

## Funding

This work was supported by grants from the Development and Reform Commission of Jilin Province (2021C042-3), the National Natural Science Foundation of China (81773317), the Science and Technology Department of Jilin Province (20180101110JC and 2018SCZWSZX-017), and the Education Department of Jilin Province (JJKH20201075KJ).

## Conflict of Interest

The authors declare that the research was conducted in the absence of any commercial or financial relationships that could be construed as a potential conflict of interest.

## Publisher’s Note

All claims expressed in this article are solely those of the authors and do not necessarily represent those of their affiliated organizations, or those of the publisher, the editors and the reviewers. Any product that may be evaluated in this article, or claim that may be made by its manufacturer, is not guaranteed or endorsed by the publisher.
